# Effect of Exercise Training on Lipoprotein Subclass Particle Concentrations and Sizes in Older Women: Results from a Randomized Controlled Trial

**DOI:** 10.3390/geriatrics8060116

**Published:** 2023-11-29

**Authors:** Ryan R. Porter, Joshua R. Sparks, J. Larry Durstine, Sabra S. Custer, Raymond W. Thompson, Xuewen Wang

**Affiliations:** 1 Department of Kinesiology, Texas Christian University, Fort Worth, TX 76129, USA; r.porter@tcu.edu; 2 Reproductive Endocrinology and Women’s Health Laboratory, Pennington Biomedical Research Center, Louisiana State University, Baton Rouge, LA 70803, USA; joshua.sparks@pbrc.edu; 3 Department of Exercise Science, University of South Carolina, Columbia, SC 29208, USA; ldurstin@mailbox.sc.edu (J.L.D.); rwthomps@mailbox.sc.edu (R.W.T.); 4 College of Nursing, University of South Carolina, Columbia, SC 29208, USA; sabra.custer@sc.edu

**Keywords:** exercise training, lipoprotein subclass, lipoprotein particle, older women

## Abstract

Background: Evidence suggests that lipoprotein subclass particles are critical markers of cardiovascular disease (CVD) risk. Older women have increased CVD risk related to age. The purpose of this study was to determine whether low and moderate doses of exercise influence lipoprotein subclasses. Methods: Women (60–75 years) were randomized into groups for 16 weeks of moderate-intensity exercise training at a low or moderate dose (33.6 and 58.8 kJ/kg body weight weekly, respectively). Lipoprotein subclasses were determined by nuclear magnetic resonance spectroscopy before and after the training. RESULTS: The average weekly exercise duration was 109 and 164 min, for low- and moderate-dose groups, respectively. In the low-dose group, high-density lipoprotein particle (HDL-P) concentration decreased (Δ = −1.9 ± 3.1 µmol/L, mean ± SD, *p* = 0.002) and mean HDL-P size increased (Δ = 0.1 ± 0.3 nm, *p* = 0.028). In the moderate-dose group, mean HDL-P size (Δ = 0.1 ± 0.2 nm; *p* = 0.024) and low-density lipoprotein particle size increased (Δ = 0.4 ± 3.9 nm; *p* = 0.007). Baseline body mass index, peak oxygen consumption and age were associated with changes in a few lipoprotein subclasses. Conclusions: In this sample of inactive older women, moderate-intensity exercise training at a dose equivalent to or even lower than the minimally recommended level by public health agencies induced changes in lipoprotein subclasses in line with reduced CVD risk. However, higher doses are encouraged for greater health benefits.

## 1. Introduction

Cardiovascular disease (CVD) is the leading cause of mortality and accounts for 23% of adult deaths in the United States [1]. According to a 2016 report by the American Heart Association (AHA), 69% of men and 68% of women 60–79 years of age and 85% of men and 86% of women 80+ years of age have CVD [2]. Though many different factors have been utilized to determine CVD risk, blood lipid and lipoprotein cholesterol concentrations are among the most established and universally accepted CVD risk determinants [3,4,5]. Traditional blood lipid and lipoprotein profiles include triglycerides (TG), total cholesterol (TC), high-density lipoprotein cholesterol (HDL-C), low-density lipoprotein cholesterol (LDL-C), and the ratio of TC to HDL-C. Predicting CVD risk through the investigation of blood lipoprotein particle concentration and size has become more readily available with the advent of technological assessment beyond traditional lipid and lipoprotein profiles [6]. Prior studies suggest that lipoprotein subclasses are critical markers of CVD risk independent of lipid and lipoprotein cholesterol [7,8,9,10,11,12,13,14,15,16].

Exercise is well recognized for its health benefits, including its reduction of CVD risk [17]. Earlier studies indicate that a dose-response relationship between exercise and lipids and lipoprotein cholesterol exists, and noticeable desired lipid changes are likely to occur when weekly exercise accrues to 1200–2200 kcal (285–524 kJ) [18]. A few studies have demonstrated that exercise can impact lipoprotein subclasses [19,20,21,22,23]. A meta-analysis of six studies concluded that exercise training decreased the concentrations of large very low-density lipoprotein particles (VLDL-Ps), small LDL particles (LDL-Ps), medium HDL particles (HDL-Ps), and mean VLDL-P size, and increased large LDL-P and large HDL-P concentrations and mean LDL-P size [24].

Though exercise impacts lipoprotein subclasses, few studies have specifically investigated whether the dose or amount of exercise influences lipoprotein subclass particle concentrations and sizes. Kraus et al. demonstrated that middle-aged overweight and obese adults with dyslipidemia completing higher exercise training amounts had greater health improvements than those completing lower exercise training amounts concerning most lipoprotein variables, while a lower exercise amount had better responses in all variables than the control group [21].

As women age, their risk of CVD increases, and their physical fitness declines [25,26]. Therefore, exercise is likely a therapeutic means to improve women’s cardiovascular health. The doses of the exercise in the study by Kraus et al. (high amount: an average of 176 min/week at high intensity; low amount: an average of 176 min/week at moderate intensity or 117 min at high intensity) [21] were higher than the minimum recommended for the general population to achieve health benefits [27]. Due to age-related decline in physical activity and physical performance [25], participating in a higher exercise dose may be challenging for older adults, specifically older women who have not been habitually active. Thus, examining responses to an exercise dose at or even lower than the recommended level would provide useful information for public health. Therefore, the purpose of this study was to investigate the effects of 16 weeks of exercise training at a dose equivalent to the recommended minimum dose and a lower dose on the blood lipoprotein subclass profile in older women.

## 2. Methods

### 2.1. Study Population

The current secondary analysis utilized data from the Women’s Energy Expenditure in Walking Programs (WEWALK; clinicaltrials.gov registration number—NCT01722136) study [28]. The WEWALK study protocol was reviewed and approved by the University of South Carolina Institutional Review Board (IRB# Pro00016306). Prior to beginning the study, all participants signed an informed consent form. In brief, all participants of this study were female, 60–75 years of age, weight stable (±3% body weight for previous three months), inactive (no more than 20 min of structured exercise three times per week for the past three months), and non-smoking in the last year; had a body mass index (BMI) ≥ 18 and ≤30 kg/m^2^; and were free from CVD, metabolic or respiratory disease and any other condition that may affect adherence to the study protocol.

### 2.2. Exercise Intervention

Prior to commencing the exercise intervention, participants were randomized to one of two moderate-intensity treadmill-walking groups that differed by exercise dose, defined by weekly exercise energy expenditure. Age and BMI were considered in randomization so that for every two participants in the same categories of age (60–64.9, 65–69.9, and 70–75 years) and BMI (<25 and ≥25 kg/m^2^), one was allocated into each group using a list of random numbers generated using Research Randomizer (www.randomizer.org). The low-dose group and the moderate-dose group were prescribed an exercise dose of 8 kilocalories (33.6 kJ) and 14 kilocalories (58.8 kJ) per kilogram of body weight per week, respectively. Weekly target energy expenditure was determined by multiplying the participant’s weight by their assigned dosage. The two different doses were achieved by varying the total duration of exercise completed in a week. Three to four exercise sessions per week were prescribed. The target exercise intensity was 50–55% of heart rate reserve, which was calculated using resting heart rate and the peak heart rate achieved during the graded exercise test at baseline (see below).

All training sessions were supervised in a clinical exercise research setting. The exercise intensity and weekly duration were incrementally increased. The target intensity and dose were reached by week five in the low-dose group and week eight in the moderate-dose group. A 3 min warm-up and cool-down was conducted for each exercise session. Heart rate monitors (FT1; Polar, Lake Success, NY, USA) were utilized to continuously monitor exercise training intensity. If the heart rate was out of range, exercise intensity was adjusted. Blood pressure was measured before, at the mid-point, and after each exercise session.

All participants were provided individualized exercise prescriptions. Small incentives, such as t-shirts and water bottles, were given to assist with participant retention. Certificates for participation were provided to selected participants each month, such as “best attendance” and “best effort”. All participants were instructed to put a ticket in a bottle each time they attended exercise training in our research center. A winning ticket was drawn from the bottle each month and the specific participant was given a USD 20 prize. Monetary compensation was additionally provided for completing baseline measurements (USD 50) and post-intervention measurements (USD 50).

### 2.3. Measurements

#### 2.3.1. Body Mass Index (BMI)

Height and weight were measured to the nearest 0.1 cm and 0.1 kg, respectively. Body weight was measured with participants wearing standard scrubs, without shoes or other outer garments on a digital scale (Health O Meter^®^ 10 Professional, Pelstar LLC, McCook, IL, USA) that was calibrated annually (CC Vaughan & Sons, Incorporated, Columbia, SC, USA). Two consecutive height and weight measurements were averaged and utilized to calculate BMI (kg/m^2^) for each participant before and after the completion of the intervention.

#### 2.3.2. Graded Exercise Test

Treadmill-graded exercise testing was utilized to determine participants’ cardiorespiratory fitness before and after exercise training. The protocol began at 0% grade and the participant’s self-selected pace. Every two minutes, the incline was increased by 2%. Oxygen consumption ($\dot{V}$O_2_) was continuously measured utilizing a metabolic cart (TrueOne 2400; ParvoMedics, Sandy, UT, USA). Blood pressure was measured at rest and in the last 30 s of every exercise stage utilizing a stethoscope and sphygmomanometer. Participants were encouraged to continue exercising to volitional fatigue. During the test and for 10 min following the test, heart rhythm was monitored by a health care professional utilizing a standard 12-lead electrocardiogram (ECG) (Q-Stress^®^; Cardiac Science, Bothell, WA, USA). Test results were considered peak if at least two of the following four criteria were met: a plateau of $\dot{V}$O_2_ (within 2 mL/kg/min in last 2 min), a maximal heart rate greater than 90% of age-predicted maximal HR (HRmax) (220–age), a self-reported rating of perceived exertion greater than 17 on the 6–20 Borg scale, and/or a respiratory exchange ratio greater than or equal to 1.10. Peak oxygen consumption ($\dot{V}$O_2_peak) was determined by the highest 30 s $\dot{V}$O_2_ average recorded during the test.

#### 2.3.3. Blood Sample Collection and Lipoprotein Subclass Measurements

Blood samples were collected following an overnight fast at baseline and after the 16-week exercise training. The post-intervention blood sample was collected at least 24 h after but within 7 days of the last exercise training session. The median cubital or cephalic vein in the cubital fossa of the elbow was used to collect blood unless these veins were compromised. Blood was collected into a vacutainer^®^ EDTA tube and centrifuged at 3000 rpm to separate the blood cells from plasma. Plasma was then aliquoted and stored at −80 °C until analysis.

Plasma samples were analyzed by nuclear magnetic resonance (NMR) spectroscopy at LipoScience, Inc. (Raleigh, NC, USA), using their proprietary NMR platform [6]. The inter-assay and intra-assay coefficients of variation were between 2.6 and 5.8% for LDL-P [29] and between 2.0 and 3.9% for HDL-P [30]. Each measurement provides concentrations of large VLDL and chylomicron particles, medium and small VLDL-Ps and HDL-Ps, large and small LDL-Ps, and intermediate-density lipoprotein particles (IDL-Ps), as well as weighted-average VLDL-P, LDL-P, and HDL-P sizes. The weighted average particle diameter for each lipoprotein is calculated as the sum of the lipoprotein subclass diameters multiplied by its relative mass percentage as estimated from the amplitude of its methyl NMR signal. Total VLDL-P, LDL-P, and HDL-P concentrations were calculated as the sum of their respective subclass concentrations. Total TG, VLDL, chylomicron TG, and HDL-C concentrations were calculated and provided by LipoScience, Inc. (Raleigh, NC, USA).

## 3. Statistics

Descriptive statistics were calculated and reported as means and standard deviations (SDs) for each exercise intervention group. Independent sample t-tests or Chi-square tests were utilized to determine differences in baseline characteristics between exercise groups. General linear models with repeated measures, including a group-by-time interaction, were utilized to determine if any variable changed differently between the two groups following the respective interventions. When the interaction term was not statistically significant, the two intervention groups were combined to determine whether there was a significant effect of exercise training regardless of exercise dose. We were interested in the effects of both exercise interventions; therefore, general linear models were also used to determine changes in each group. In further analyses, age, BMI, and $\dot{V}$O_2_peak at baseline were adjusted.

Lastly, we performed linear regression analyses to determine the associations of baseline age, BMI, and $\dot{V}$O_2_peak, with changes in lipoprotein subclasses, in order to understand whether baseline characteristics influenced responses to the exercise intervention. The changes in lipoprotein subclasses were calculated using values after interventions minus at baseline. Baseline age, BMI, and $\dot{V}$O_2_peak were examined as both continuous and categorical variables: age (<64.5 years or ≥64.5 years), BMI (normal weight or overweight), and $\dot{V}$O_2_peak (low <20.2 mL/kg/min, or high: ≥ 20.2 mL/kg/min). Statistical significance was set at *p* < 0.05. All analysis was performed utilizing SAS 9.4 (SAS Institute Inc., Cary, NC, USA).

## 4. Results

### 4.1. Participant Characteristics

A total of 65 participants (35 in the low-dose and 30 in the moderate-dose group) completed the exercise intervention and had lipoprotein subclass data at both baseline and post-intervention. In the parent trial, 87 participants were randomized and began exercise intervention and 72 completed the study [28]. However, blood samples were not available at baseline and/or post-intervention for seven participants who completed the study; therefore, these participants were not included in this analysis. These seven participants were not different from those who had complete lipoprotein data in age, body weight, BMI, or $\dot{V}$O_2_peak at baseline.

Participant characteristics are presented in Table 1. The average age of the participants was 65.1 ± 4.2 years, and they were mostly white (86%). No significant differences were found between the two exercise groups for age, racial/ethnic distribution, education, income, employment, marital status, body weight, BMI, or $\dot{V}$O_2_peak at baseline. As by trial design, the average heart rate during exercise sessions was similar between the two groups, and weekly exercise duration was longer in the moderate-dose compared to the low-dose group.

Body weight, BMI, and $\dot{V}$O_2_peak after exercise interventions are also included in Table 1. The two groups did not change differently in body weight or BMI from baseline to post-intervention (p for group x time interaction = 0.988 and 0.935, respectively). Body weight significantly decreased by 0.8 ± 2.1 kg (*p* = 0.007), and BMI significantly decreased by 0.3 ± 0.8 kg/m^2^ (*p* < 0.001) in the overall sample. A significant group x time interaction was observed for change in $\dot{V}$O_2_peak from baseline to post-intervention (*p* = 0.029). Specifically, $\dot{V}$O_2_peak significantly increased in the moderate-dose group by 2.4 ± 3.2 mL/kg/min (*p* < 0.001) while remaining unchanged in the low-dose exercise group after intervention (*p* = 0.15).

### 4.2. Lipoprotein Subclass

Table 2 includes lipoprotein subclass particle concentrations and sizes at baseline and post-intervention. At baseline, no differences between the two exercise groups in any of these measures (0.155 ≤ *p* ≤ 0.916 for all) existed. No significant differences between the two exercise groups were found for changes in any lipoprotein subclass particle concentration or size (*p* values for group x time interaction range: 0.062–0.994). 

Mean HDL-P size increased in both the low-dose (Δ = 0.1 ± 0.3 nm, *p* = 0.028) and moderate-dose group (Δ = 0.1 ± 0.2 nm; *p* = 0.024), resulting in an increase in the overall sample (Δ = 0.1 ± 0.3 nm; *p* = 0.002). Total HDL-P concentration decreased in the low-dose exercise group (Δ = −1.9 ± 3.1 µmol/L; *p* = 0.001) and did not significantly change in the moderate-dose group, but it significantly decreased in the overall sample (Δ = −1.5 ± 3.6 µmol/L; *p* = 0.002). LDL-P size increased by 0.4 ± 3.9 nm (*p* = 0.007) in the moderate-dose group only. Small VLDL-P concentration decreased in the total sample (Δ = −4.2 ± 16.4 nmol/L; *p* = 0.041) but did not reach statistical significance in either group alone.

Additionally, among the measures calculated by NMR, TG concentration had a different change in the moderate-dose versus the low-dose group (p for group x time interaction = 0.020). TG concentration significantly decreased in the low-dose group (Δ = −2.2 ± 34.6 mg/dL; *p* = 0.02) but did not change in the moderate-dose group or in the overall sample (*p* ≥ 0.09 for both).

No other significant changes were observed for any other lipoprotein subclass variables or NMR calculated lipids in any group alone or in the overall sample. Adjusting for baseline age, BMI, and $\dot{V}$O_2_peak did not change these results.

### 4.3. Baseline BMI, $\dot{V}$O_2_peak, and Age as Predictors of Changes in Lipoprotein Subclasses

We further sought to elucidate potential baseline predictors of changes in lipoprotein subclasses after intervention. Table 3 presents slopes from regression models using BMI, $\dot{V}$O_2_peak, and age as continuous and categorical predictors. BMI was stratified according to standard cutoff: normal weight:18.5–24.9 kg/m^2^, and overweight: 25.0–29.9 kg/m^2^, while $\dot{V}$O_2_peak and age were stratified using their respective median of the total sample (median $\dot{V}$O_2_peak = 20.2 mL/kg/min and median age = 64.5 years).

BMI at baseline was associated with changes in medium VLDL-P concentration, with each unit of higher BMI at baseline associated with a 0.82 nmol/L less change in medium VLDL particle concentration (*p* = 0.01). Compared to those at a normal weight at baseline, women overweight at baseline had a smaller increase in medium VLDL-P concentration (difference in mean change: overweight versus normal weight: −6.18 nmol/L, *p* = 0.01). Similarly, each 1 mL/kg/min higher $\dot{V}$O_2_peak at baseline was associated with a 0.12 µmol/L greater change in large HDL-P concentration. Women with a higher $\dot{V}$O_2_peak at baseline had a greater increase in large HDL-P concentration than women with a low $\dot{V}$O_2_peak (difference in mean change: high versus low $\dot{V}$O_2_peak: 0.91 µmol/L, *p* = 0.03). Additionally, compared to women <64.5 years at baseline, women ≥64.5 years had greater mean change (difference versus <64.5 years: 9.99 nmol/L, *p* = 0.04) in total VLDL and chylomicron-P concentration. This association, however, was not significant when examining age as a continuous variable (*p* = 0.13). No other significant associations of baseline BMI, $\dot{V}$O_2_peak, and age with changes in lipoprotein subclasses were found.

## 5. Discussion

We investigated lipoprotein subclass changes in response to two moderate-intensity exercise interventions in inactive older women. The moderate-dose intervention was equivalent to the minimum exercise dose recommended for the general population [27]. The low dose, however, was lower than the recommended level. A few lipoprotein subclass variables significantly changed within each exercise group, indicating that older women participating in exercise, even at a lower amount, with minimal weight change, may experience changes in lipoprotein profile.

In our study, no between-group differences were observed for any blood lipoprotein subclass. These findings are inconsistent with the findings of Kraus et al., who found that moderate-dose exercise elicited a greater change in some blood lipoprotein subfractions (large VLDL-P, IDL-P, total and small LDL-P, and large HDL-P concentration; mean LDL-P and HDL-P size) compared to low-dose exercise [21]. Several study design differences between their study and our study could contribute to the inconsistent findings and smaller magnitude of change in lipoprotein subclasses in our study. First, their participants had mild-to-moderate dyslipidemia, while participants in our study were generally without dyslipidemia. Second, the weekly exercise doses in their study were higher than those in our study. Their exercise groups included a low amount at a moderate intensity of an average of 176 min/week, a low amount at a high intensity of 117 min/week, and a high amount at a high intensity of 176 min/week. In contrast, our study included a low-dose group exercising for an average of 109 min/week and a moderate-dose group exercising for 164 min/week, both at moderate intensity. Thus, our moderate-dose exercise was even slightly lower than the low-amount exercise of Kraus et al. Third, the exercise training lasted 16 weeks in our study and 6 months in their study, resulting in a difference in the total amount of exercise over the entire study. Furthermore, our study was comprised of non-obese women, 60–75 years of age, while Kraus et al. included overweight and obese men and women, 40–65 years of age. Age, sex, and BMI status could also influence the results, as supported by our analyses of associations of baseline characteristics with changes in blood lipoprotein subclasses.

Though our study found no significant differences between groups after the exercise intervention, multiple variables did significantly change within each exercise group and in the overall sample, indicating that exercise did influence blood lipoprotein subclasses. The decrease in total HDL-P concentration in the low-dose group and overall sample could be viewed as a less favorable outcome because HDL-P is inversely associated with CVD risk [31]. However, one must consider the entire HDL-P profile. While total HDL-P concentration decreased, mean HDL-P size increased in both training groups and when grouped together. This increase in HDL-P size has been noted in other studies and is viewed as favorable, as this change is indicative of a greater cholesterol-carrying capacity of the circulating HDL particles [16,32,33,34] and may, in part, explain the decreased HDL-P concentrations. The same rationale can also apply to the increase in LDL-P size observed in the moderate-dose exercise group [35]. However, we cannot explain why TG concentration decreased in the low-dose group but did not change in the moderate-dose group. It should be noted that the TG concentration was calculated based on algorithms by NMR. Our previous analyses showed that the two groups were similar in measures of non-exercise activity thermogenesis, daily physical activity counts (determined by accelerometer), and time spent sedentary and in physical activity of light or moderate-to-vigorous intensities at baseline and during the last two weeks of intervention [28,36]. A meta-analysis found negligible-to-small changes in appetite control and food preference measures from pre- to post-exercise training [37]. However, we could not rule out potential changes in diet and physical activity other than the exercise sessions throughout the intervention, which could influence our outcome measures. Since the increase in LDL-P size was only observed in the moderate-dose group, which also did not have a decrease in HDL-P concentration, these data provide preliminary evidence that a higher dose of aerobic exercise training potentially provides greater benefit to the blood lipoprotein profile compared to lower-dose aerobic exercise training.

BMI, cardiorespiratory fitness, and age are crucial determinants of health when examining health span in adults and are shown to be predictors of adherence to previous behavioral interventions [38,39]. Present evidence supports that a higher BMI is positively associated with less desirable blood lipoprotein and lipid levels (e.g., lower HDL-C, and higher LDL-C and TC) [40], while higher cardiorespiratory fitness is associated with more favorable traditional blood lipoprotein profiles (e.g., higher HDL-C, and lower LDL-C and TC) [41]. However, whether baseline BMI, cardiorespiratory fitness, and age are associated with changes in blood lipoprotein subclasses with exercise intervention has yet to be elucidated. Our study findings, although preliminary, suggest that BMI, cardiorespiratory fitness, and age at baseline may influence changes in lipoprotein subclasses. Further research is needed to elucidate the implications of the baseline characteristics in predicting responses to treatments, which will contribute to more personalized interventions.

The strengths of this study include the use of NMR spectroscopy to analyze lipoprotein particle concentrations and sizes, and the exercise intervention sessions were supervised in a clinical exercise setting to assure protocol compliance. The generalizability of this study is limited to generally healthy, older women. The absence of a control group for comparison is a limitation of the study. Additionally, we did not monitor diet or non-exercise physical activity throughout the study.

In conclusion, the exercise intervention induced changes in lipoprotein subclasses. Although the intervention-induced changes are less than those in previous studies, our results indicate that inactive older women participating in exercise at the recommended dose for the general population, or even lower than that, could still experience changes in blood lipoprotein subclasses that are in line with reduced CVD risk. However, it is important to note that in order to receive greater health benefits, higher doses of physical activity/exercise are encouraged.

## Figures and Tables

**Table 1:** Participant characteristics at baseline and post-intervention.

	Overall (*n* = 65)	Low Dose (*n* = 35)	Moderate Dose (*n* = 30)
Age, years	65.1 ± 4.2	65.6 ± 4.7	64.6 ± 3.6
Race, *n* (%)			
White	56 (86.2)	32 (91.4)	24 (80.0)
Black	8 (12.3)	3 (8.6)	5 (16.7)
Hispanic	1 (1.5)	0 (0.0)	1 (3.3)
Education, *n* (%)			
<College Graduate	22 (33.8)	11 (31.4)	11 (36.7)
≥College Graduate	42 (64.6)	23 (65.7)	19 (63.3)
Missing	1 (1.6)	1 (2.9)	0 (0.0)
Income, *n* (%)			
<$50,000 USD/year	18 (27.7)	9 (25.7)	9 (30.0)
≥$50,000 USD/year	46 (70.7)	25 (71.4)	21 (70.0)
Missing	1 (1.6)	1 (2.9)	0 (0.0)
Employment, *n* (%)			
Employed	32 (49.2)	19 (54.3)	13 (43.3)
Unemployed/Retired	32 (49.2)	15 (42.8)	17 (56.7)
Missing	1 (1.6)	1 (2.9)	0 (0.0)
Marital Status, *n* (%)			
Married	38 (58.4)	20 (57.1)	18 (60.0)
Not Married/Widowed	26 (40.0)	14 (40.0)	12 (40.0)
Missing	1 (1.6)	1 (2.9)	0 (0.0)
Body Weight, kg Baseline Post-Intervention	67.5 ± 9.7 66.7 ± 9.5 ^b^	67.4 ± 10.1 66.7 ± 10.0	67.6 ± 9.3 66.8 ± 9.2
Body Mass Index, kg/m^2^			
Baseline	25.6 ± 3.6	25.8 ± 4.0	25.5 ± 3.1
Post-Intervention	25.3 ± 3.6 ^c^	25.4 ± 4.0 ^a^	25.1 ± 3.1 ^b^
$\dot{V}$O_2_peak, mL/kg/min			
Baseline	20.4 ± 3.7	20.4 ± 3.7	20.4 ± 3.7
Post-Intervention	21.9 ± 4.7 ^c^	21.1 ± 4.3	22.8 ± 5.1 ^c^
Exercise Sessions			
Heart Rate, beats/min		114.2 ± 10.8	115.9 ± 10.2
% Heart Rate Reserve, %		48.5 ± 11.5	48.7 ± 12.2
Duration, minutes/week		108.5 ± 9.1	163.5 ± 12.6
Actual/Prescribed Exercise Dose, %		105.1 ± 8.1	98.9 ± 7.7

$\dot{V}$O_2_peak, peak volume of oxygen consumption during graded exercise test. Compared to respective baseline: ^a^, *p* < 0.05; ^b^, *p* < 0.01; ^c^, *p* < 0.001.

**Table 2:** Lipoprotein subclass profile at baseline and post-intervention measured by nuclear magnetic resonance (NMR) spectroscopy.

	Overall (*n* = 65)		Lower Dose (*n* = 35)		Higher Dose (*n* = 30)	
	Baseline	Post-Intervention	Baseline	Post-Intervention	Baseline	Post-Intervention
VLDL and Chylomicron Particle, nmol/L						
Total	49.1 ± 20.0	45.3 ± 17.7	48.8 ± 18.2	42.6 ± 15.0	49.5 ± 22.3	48.5 ± 20.2
Large VLDL and Chylomicron-P	3.8 ± 2.9	3.6 ± 3.6	4.1 ± 3.0	3.2 ± 3.2	3.6 ± 2.8	4.0 ± 3.9
Medium VLDL-P	14.7 ± 9.7	15.3 ± 9.8	14.6 ± 9.8	14.4 ± 8.8	14.8 ± 9.8	16.4 ± 10.9
Small VLDL-P	31.0 ± 14.4	27.2 ± 12.6 ^a^	31.0 ± 12.7	26.4 ± 14.0	31.1 ± 16.4	28.1 ± 11.1
IDL and LDL Particle, nmol/L						
Total	1034.4 ± 290.5	1020.8 ± 318.5	1016.0 ± 325.5	1015.1 ± 355.3	1055.9 ± 247.2	1046.9 ± 274.3
IDL-P	274.7 ± 147.9	282.6 ± 133.0	270.7 ± 143.3	303.7 ± 122.8	279.4 ± 155.5	258.0 ± 142.1
Large LDL-P	281.8 ± 195.2	303.4 ± 196.0	307.4 ± 204.7	315.0 ± 215.3	252.8 ± 183.0	290.2 ± 174.4
Small LDL-P	490.8 ± 300.4	448.6 ± 371.1	455.5 ± 320.5	405.5 ± 405.7	532.0 ± 274.8	495.8 ± 325.8
HDL Particle, µmol/L						
Total HDL-P	38.8 ± 5.9	37.4 ± 5.5 ^b^	39.4 ± 5.6	37.6 ± 5.6 ^c^	38.1 ± 6.3	37.2 ± 5.5
Large HDL-P	9.9 ± 3.2	10.2 ± 3.4	10.2 ± 3.7	10.5 ± 3.7	9.7 ± 2.4	9.8 ± 3.0
Medium HDL-P	13.7 ± 8.0	12.8 ± 7.3	14.6 ± 7.5	13.3 ± 7.0	12.8 ± 8.7	12.1 ± 7.7
Small HDL-P	15.6 ± 7.6	15.2 ± 8.3	15.1 ± 8.1	14.5 ± 9.0	16.2 ± 7.1	16.2 ± 7.4
Particle Size, nm						
VLDL-P	49.4 ± 7.0	49.2 ± 7.1	50.5 ± 7.6	49.9 ± 7.1	48.0 ± 6.1	48.3 ± 7.1
LDL-P	20.7 ± 0.6	20.8 ± 0.7	20.8 ± 0.6	20.8 ± 0.7	20.6 ± 0.5	20.9 ± 0.7 ^a^
HDL-P	9.7 ± 0.4	9.8 ± 0.5 ^b^	9.7 ± 0.5	9.8 ± 0.6 ^a^	9.7 ± 0.4	9.8 ± 0.5 ^a^
NMR Calculated Lipids, mg/dL						
TG	114.8 ± 47.1	112.6 ± 52.5	116.9 ± 41.9	105.5 ± 43.5 ^a^	112.4 ± 53.3	120.9 ± 61.1
VLDL and Chylomicron TG	76.6 ± 30.8	74.8 ± 34.4	77.6 ± 27.3	70.5 ± 27.3 ^a^	75.5 ± 34.9	79.8 ± 41.2
HDL-C	68.2 ± 15.4	67.2 ± 16.0	69.9 ± 16.2	68.9 ± 17.2	66.2 ± 14.4	65.3 ± 14.4

Compared to respective baseline: ^a^, *p* < 0.05; ^b^, *p* < 0.01; ^c^, *p* < 0.001.

**Table 3:** Association between baseline body mass index, cardiorespiratory fitness ($\dot{V}$O_2_peak), and age, analyzed as continuous and categorical variables, with changes in lipoprotein subclasses from baseline to post-intervention.

	Body Mass Index		$\dot{V}$O_2_peak		Age	
	Continuous	Categorical	Continuous	Categorical	Continuous	Categorical
VLDL and Chylomicron Particle Concentration (nmol/L)						
Total	−0.10 (0.68)	−3.70 (4.91)	−0.24 (0.67)	−4.41 (4.83)	0.88 (0.57)	9.99 (4.71) ^a^
Large VLDL and Chylomicron-P	0.07 (0.10)	0.36 (0.70)	0.02 (0.09)	−0.70 (0.68)	0.00 (0.08)	−0.10 (0.69)
Medium VLDL-P	−0.82 (0.31) ^b^	−6.18 (2.25) ^b^	0.14 (0.32)	−0.16 (2.34)	0.21 (0.28)	2.56 (2.33)
Small VLDL-P	0.65 (0.57)	2.12 (4.15)	−0.40 (0.56)	−3.53 (4.07)	0.67 (0.48)	7.54 (4.00)
IDL and LDL Particle, nmol/L						
Total LDL-P	5.80 (7.31)	25.72 (53.28)	−1.25 (7.22)	−14.60 (52.59)	6.95 (6.22)	4.52 (52.77)
IDL Particles	−7.05 (5.33)	−51.99 (38.68)	1.92 (5.30)	−20.46 (38.59)	5.12 (4.57)	6.50 (38.78)
Large LDL Particles	2.22 (7.06)	13.05 (51.28)	3.18 (6.93)	6.00 (50.57)	−2.62 (6.03)	−12.77 (50.70)
Small LDL Particles	10.61 (7.75)	64.64 (56.58)	−6.36 (7.69)	−0.18 (56.35)	4.46 (6.71)	11.12 (56.49)
HDL Particle, µmol/L						
Total HDL Particles	−0.02 (0.13)	0.45 (0.92)	−0.04 (0.12)	−1.56 (0.89)	0.08 (0.11)	0.21 (0.91)
Large HDL Particles	−0.10 (0.06)	−0.43 (0.43)	0.12 (0.06) ^b^	0.91 (0.41) ^a^	0.07 (0.05)	0.14 (0.42)
Medium HDL Particles	−0.03 (0.22)	−0.96 (1.59)	0.12 (0.22)	−1.30 (1.56)	−0.15 (0.19)	−1.06 (1.57)
Small HDL Particles	0.10 (0.23)	1.82 (1.67)	−0.27 (0.22)	−1.17 (1.65)	0.17 (0.20)	1.13 (1.66)
Particle Size, nm						
VLDL Size	0.30 (0.25)	2.28 (1.83)	0.11 (0.25)	−1.80 (1.81)	−0.18 (0.22)	−2.55 (1.80)
LDL Size	−0.01 (0.02)	−0.13 (0.17)	0.02 (0.02)	−0.01 (0.17)	−0.01 (0.02)	−0.03 (0.17)
HDL Size	−0.02 (0.01)	−0.12 (0.06)	0.01 (0.01)	0.11 (0.06)	0.00 (0.01)	0.02 (0.06)
NMR Calculated Lipids, mg/dL						
TG	0.20 (1.21)	−3.85 (8.18)	0.28 (1.19)	−7.69 (8.60)	0.80 (1.03)	7.83 (8.62)
VLDL and Chylomicron TG	−0.12 (0.86)	−3.50 (6.22)	0.48 (0.84)	−6.21 (6.09)	0.71 (0.73)	6.70 (6.10)
HDL-C	−0.41 (0.25)	−1.11 (1.82)	0.43 (0.24)	1.14 (1.79)	0.31 (0.21)	0.90 (1.80)

Data are presented as estimate (standard error) from unadjusted regression models. Changes in lipoprotein subclass were calculated by subtracting baseline from post-intervention value, and they were used as dependent variables in the models. When body mass index, $\dot{V}$O_2_peak, and age were analyzed as categorical variables, the reference is normal weight (<25 kg/m^2^), low $\dot{V}$O_2_peak (< 20.2 mL/kg/min), and age < 64.5 years, respectively. ^a^, *p* < 0.05; ^b^, *p* < 0.01.

## Data Availability

Data sharing is available upon reasonable written request to the corresponding author and execution of a data sharing agreement.
